# Improving the Science of Adolescent Social Media and Mental Health: Challenges and Opportunities of Smartphone-Based Mobile Sensing and Digital Phenotyping

**DOI:** 10.1007/s41347-024-00443-5

**Published:** 2024-10-18

**Authors:** Jessica L. Hamilton, Melissa J. Dreier, Bianca Caproni, Jennifer Fedor, Krina C. Durica, Carissa A. Low

**Affiliations:** 1 Rutgers University-New Brunswick, 53 Avenue E., Piscataway, NJ 08854 USA; 2 University of Pittsburgh, Pittsburgh, PA USA

**Keywords:** Social media, Adolescence, Smartphone, Sensing, Mental health, Feasibility

## Abstract

The impact of social media (SM) use (‘screentime’) on adolescent mental health has been the focus of increasing concern, despite mixed findings from empirical research. Current methodological approaches rely on self-reported SM use, which has limited accuracy and obscure the dynamic interplay of SM use and mental health. Smartphone-based mobile sensing offers new opportunities to gain insights into adolescents’ SM use patterns and behaviors, particularly at an idiographic level. Considerations and challenges of smartphone sensing methods for capturing adolescents’ SM use patterns and behaviors in clinical psychological science are discussed in the context of a pilot study using smartphone-based sensing with adolescents. The pilot study included 19 adolescents (Mean age = 15.84; 68% boys; 79% White) who installed a passive monitoring application (AWARE) on their phones for 31 (SD = 5.6) days. Descriptive data of sensing acceptability and feasibility are presented based on participant ratings and data yield ratio of usable

data (74.18%). Sensing yielded 10,038 hourly observations collected from the ‘application foreground’ sensor across all participants from social media apps, and a total of 645 applications used. Categorization of SM apps were coded (kappa >.90) into ‘social networking’ (N = 20 apps) and ‘broader SM’ (N = 41) and compared to both Play Store-defined SM apps (N = 26) and popular SM apps based on Common Sense Media Survey (N = 9). Descriptive data on extracted behavioral features (duration, checking) from SM use categories (binned hourly and daily) are presented. Challenges, opportunities, and future directions of sensing methods for SM use are discussed to inform our understanding of its impacts on mental health and to improve the rigor of SM research in clinical psychological science.

**Supplementary Information:**

The online version contains supplementary material available at 10.1007/s41347-024-00443-5.

The impact of social media use on adolescent mental health has been the focus of increasing concern from government officials, educators, clinicians, and parents. To date, much research has focused on screentime related to social media (i.e., time spent using social media) and mental health outcomes (Odgers & Jensen, 2020), with some studies finding significant negative effects of social media (Gámez-Guadix, 2014; Twenge & Farley, 2021) and others indicating positive or null effects of social media on mental health (Coyne et al., 2020; Kreski et al., 2021; Orben & Przybylski, 2019). Yet, key methodological approaches in mental health research on this topic have reduced our ability to draw firmer conclusions about social media use and its impact on adolescent development and mental health (Valkenburg, Meier, et al., 2022a, 2022b).

Most research in mental health fields (e.g., clinical psychology) on this topic is entirely reliant on self-reported social media use or teens’ perceptions of how much they are using social media on a ‘typical day’. This is problematic as recent research indicates that self-reported estimates are unreliably associated with objective patterns of social media use (Junco, 2013; Mahalingham et al., 2023; Parry et al., 2021; Verbeij et al., 2021). Furthermore, individuals with depressive symptoms may be more likely to overestimate their social media use, thereby inflating estimates and its association with depression (Sewall et al., 2020). Second, most self-reported items ask adolescents to report social media on a ‘typical’ day, which assumes that social media use is stable over time. However, research indicates that social media use fluctuates within-person and over time (Boers et al., 2019; Siebers et al., 2022; Verbeij et al., 2021), including even a brief window of 10 days (Hamilton et al., 2021). Thus, our current understanding of social media use is largely limited to adolescents’ perceptions of their ‘typical’ social media use rather than actual social media use on a day-to-day basis, which begs the question: what do we really know about adolescents’ *actual* social media use?

## Moving Beyond Self-Reported Social Media Use to Objectively Captured Social Media

Given this limitation in our current measurement and assessment of social media use, researchers have employed methods to objectively capture social media use through teens’ social media accounts and smartphones such as through social media data donations (van Driel et al., 2022) and “screenomics” (e.g., obtaining near-continuous screenshots of individuals content and behaviors on their personal devices; Brinberg et al., 2021; Jacobucci et al., 2024). While access to social media content provides valuable information about teens’ social media behaviors (e.g., engagement) and types of content engaged with on social media, it is accompanied by a host of ethical, technical, and practical considerations. For instance, often data donations provide social media content for specific platforms such as Instagram (Razi et al., 2022; van Driel et al., 2022) or only capturing certain engagement (post/liking) activities rather than passive scrolling behaviors linked to mental health (Roberts & David, 2023), which limits a more comprehensive picture of teens’ overall social media use. There also are ethical concerns and data filtering challenges of receiving data with identifying content of teens who did not consent or viewing potentially harmful or illegal content among minors. While a full review of these methods is beyond the scope of the present article (Griffioen et al., 2020), these challenges limit the scalability of social media content analysis using such approaches. Furthermore, these approaches still do not provide information about teens’ social media patterns of use, which may yield more modifiable targets for intervention.

To obtain social media screentime using more objective metrics, researchers have developed creative approaches such as having participants upload screenshots of smartphone screentime from teens’ phones (e.g., Gower & Moreno, 2018; Ohme et al., 2021; Rosenthal et al., 2021). This approach dissects information about ‘social media’ in addition to other screentime activities, thereby providing researchers with objective metrics about a single day (or repeated uploads for daily information) about screentime. There are clear strengths to this approach in understanding social media use beyond self-reported social media screen time perceptions, including objective indices and multiple reports from a person over several days or weeks, pending study design. However, there are also several drawbacks. First, this method requires active participation from adolescents to upload the screenshots (Gower & Moreno, 2018), which may result in lower compliance and reduced access to individuals’ data. Second, it relies on operating system determinations of what is defined as ‘social media’, which, as discussed below, may not accurately categorize apps. Third, it provides a snapshot of social media use in a given day based on timing of the ‘screenshot’, without the necessary depth or detail of social media *within* a day, such as which social media apps are being used, when, for how long, and how often.

## Digital Phenotyping and Mobile Sensing Using Smartphones to Understand Adolescents’ Social Media Use

In recent years, researchers have called for digital phenotyping as an approach to collect rich moment-to-moment phenotypes of digital behaviors to better understand the relationship of social media use and mental health (Perlmutter et al., 2024). Mobile sensing offers new opportunities to gain insights into adolescents’ social media use patterns and behaviors, which may differentially impact youth mental health. Mobile sensing includes passively collecting data from participants with minimal interaction or awareness using smartphones or other devices, thereby providing continuous high-dimensional data that can capture social media use patterns (Orr et al., 2023; Zhang et al., 2023). Smartphone-based mobile sensing may be particularly relevant, given that the majority of adolescents report using social media on their smartphone (Anderson et al., 2023). Beyond total daily social media ‘screentime’ (i.e., duration of total social media use within a given day or time period), we can stay abreast of emerging social media apps that teens are using, which apps are being used and when, daily patterns of different social media applications, and frequency of checking apps—including microbursts (Ferreira et al., 2014; Lind et al., 2023). As adolescents keep the app passively installed on their phones, patterns may emerge that can be captured over days, weeks, or even months and potentially mapped onto higher level behaviors (e.g., social engagement/withdrawal) or clinical outcomes (i.e., digital phenotyping (Perlmutter et al., 2024). Digital phenotyping is defined as “the moment-by-moment quantification of the individual-level human phenotype in situ using data from personal digital devices” (Torous et al., 2016). Creating rich moment-to-moment phenotypes of digital behaviors, including social media, in relation to other important indices can yield novel insights and advance our understanding of social media and its impacts. For instance, we can evaluate hourly or daily social media use patterns (e.g., duration, frequency, timing, and variability) and predict daily behaviors or symptoms as well as clinically focused outcomes for rare events or occurrences (e.g., suicide attempts) using these approaches. Smartphone-based mobile sensing can capture social media use patterns without obtaining user content (e.g., what they post on social media, what they send in messages), thereby balancing the need for objective behavioral data with adolescents’ privacy.

Smartphone-based mobile sensing has been increasingly applied by researchers to this end. Researchers have applied mobile sensing methods to gather rich data on children’s and adolescents’ phone and technology usage (Madigan et al., 2019; Orr et al., 2023; Wade et al., 2021). Increasingly, digital phenotyping approaches are pairing passively captured data (e.g., smartphone-based mobile sensing) with actively captured and subjectively reported data (e.g., experience sampling) to understand the complex interplay between behaviors (e.g., phone use behaviors, rest-activity rhythms, etc.) and complex symptomology (e.g., mental and physical health concerns). Within the mental health field in particular, digital phenotyping has been used to understand relationships between adult smartphone use and other mental and physical health symptoms, like depression, anxiety, health, and wellbeing (Cornet & Holden, 2018; Meyerhoff et al., 2021; Moura et al., 2023; Trifan et al., 2019). However, to our knowledge, very little research to date has focused specifically on using mobile sensing to investigate adolescents’ social media usage (versus smartphone use or ‘screen time’ more broadly) nor has this research directly linked these data to adolescents’ mental health symptoms or behaviors (Harari et al., 2017). Applying mobile sensing, particularly using smartphones, and digital phenotyping to research questions on adolescent social media use and mental health outcomes has the potential to greatly impact our understanding of these relationships.

## Considerations and Challenges in Smartphone-Based Mobile Sensing for Objective Social Media Use

The potential promise of smartphone-based mobile sensing and digital phenotyping methods for capturing teens’ social media use patterns and behaviors is linked with several important considerations and challenges. First, given very few studies to date have specifically paired smartphone-based mobile sensing of social media data and sensitive mental health information among adolescents, critical questions are raised about acceptability and feasibility, or simply: Can we do this with teens? We need to understand: (1) teens’ perceptions that the study application is an invasion of privacy, particularly related to capturing social media use, (2) teens’ concerns about battery use and drainage or other technical problems, and (3) are teens willing (and able) to keep the study application open on their phones to passively collect the sensing data on their phones (e.g., how much data can we get?). Furthermore, smartphone-based mobile sensing produces high-dimensional data unique to each individual, which means human-based decisions are needed about how best to process and analyze application data in the digital phenotyping process. For instance, which time frames are most relevant (e.g., hourly, daily, timing of day), how do we define social media (e.g., which apps are included in the elusive definition of ‘social media’?), and which features are most important to capture (e.g., duration, checking frequency)? These considerations and challenges of using smartphone applications to passively capture adolescents’ SM use patterns and behaviors are discussed in the context of a pilot study with adolescents. In this study, we present preliminary data about the feasibility and acceptability of using smartphone-based mobile sensing to capture social media use with teens, as well as empirical data to define, process, and analyze objective social media use to inform the challenges, opportunities, and next steps of applying these methods.

## The Current Study

The current study, described in further detail in the “Pilot Study Method: Smartphone-based Mobile Sensing with Adolescents” section, aimed to test the feasibility and acceptability of using smartphone-based mobile sensing specifically to capture social media app use data among adolescent participants in addition to daily diary questions about mental health symptoms (depression, mood, and emotions). This study was highly exploratory in nature; however, we specified hypotheses related to the acceptability and feasibility of using smartphone-based mobile sensing among adolescents. We hypothesized that adolescents would find the smartphone-based mobile sensing application acceptable to use. We also hypothesized that use of the mobile sensing app would be feasible among adolescents, yielding an acceptable quantity of data for analysis after a one-month period.

In addition, we aimed to use an existing research-based definition of social media (Carr & Hayes, 2015, p. 51) to highlight the broad array of applications that fit this category. We then compared how smartphone applications identified by our mobile sensing app would be categorized based on this definition (including several categories) to how it would be categorized by default methods (e.g., social media as defined by the Google Play store) or popularized definitions of common social media. We hypothesized that mobile sensing data would allow for a broader range of applications to be captured and analyzed relative to pre-determined categories, as determined by the Google Play store or popularized definitions.

Findings from the study are meant to provide a template for other researchers interested in investigating the link between adolescent social media use and mental health behaviors and argues for the power of smartphone-based mobile sensing and digital phenotyping to advance this field of research (see Perlmutter et al., 2024 for review on this topic).

## Pilot Study Method: Smartphone-Based Mobile Sensing with Adolescents

### Participants and Procedure

Our pilot study took place between March and November 2020 (i.e., the early COVID lockdown period). Participants included adolescents (age 13–18; grades 9–12) who were recruited from an online registry with an online screening portal sponsored by [DEIDENTIFIED FOR REVIEW]. Adolescents and their parents interested in the study completed an online eligibility questionnaire. Participants were eligible if they were between the ages of 13 and 18 and in high school (grades 9–12), used their own Android smartphone to access social media, and were fluent in English. Among other mobile sensing applications available (e.g., Domoff et al., 2021; Lind et al., 2023; Wade et al., 2021), we chose to use the AWARE app, an open-source mobile sensing application, because there is reproducible pipeline for processing AWARE data. That said, we expect that other similar mobile sensing applications would yield similar findings to those described in this study. Participants could not use iPhones for this study because we were interested in collecting social media application usage data specifically, and iOS does not permit passive collection of these data using external mobile sensing applications. That said, researchers who are interested in other indices (e.g., call and text messaging data, light, battery, etc.) may collect data using AWARE with participants who use iPhones.

Participants who met inclusion criteria were contacted to complete a Zoom meeting with study staff, where they completed the informed consent (if participants were 18) or informed parental consent and participant assent (if participants were 13–17). After consent/assent procedures, participants completed a baseline questionnaire (e.g., demographics) and downloaded the study application (AWARE) to capture social media use. AWARE passively collects multiple sensors (e.g., accelerometer, communication (messages/calls), and screen (on/off)), but ‘applications foreground’ is the primary sensor used to sense application usage patterns (Ferreira et al., 2015). Participants were instructed to keep the application open in the background of their phone to continuously collect data. Study staff monitored incoming data daily. If a participant was missing incoming data for an extended period (approximately 24–48 h), study staff reached out to that participant to remind them to keep AWARE running in the background. The study length was approximately one month (31 days). At the end of the study, participants completed a brief exit interview with study staff to discuss their experiences with AWARE in the study. All study procedures were approved by the University Institutional Review Board.

### Study Measures

#### Participant Demographics

Participants first completed a baseline questionnaire including demographics (e.g., age, race, ethnicity, sex, gender identity, transgender status, sexual orientation, and socioeconomic status. The MacArthur Subjective Social Status Scale – Youth Version (Goodman et al., 2001) measured socioeconomic status. This measure provides participants with a visual ladder with ten rungs ranging from 1 (worse off) to 10 (best off). Participants then rate their perceived socioeconomic status on the ladder relative to society and relative to others in their school. This method of rating socioeconomic status has been shown to be more predictive of associated health outcomes relative to other methods among adolescents (Goodman et al., 2001).

#### Exit Interview: Acceptability

After the 4-week study period, participants completed a semi-structured exit interview to discuss their experiences in the study, including using the AWARE application. They were asked to report on a 1–4 Likert-scale (1 “not at all”, 2 “somewhat”, 3 “often”, 4 “almost always”) about their experiences with AWARE, including how often they had concerns about their privacy, noticed it on their phones, perceptions that it drained their battery, as well as how often the application “crashed” or sent error messages. Participants also were invited to provide additional feedback they had about AWARE (open-ended question), including frequency of crashes, or other issues and concerns they had about it. To evaluate acceptability, mean scores of acceptability ratings were calculated for each of the indices above. Frequencies/percentages were calculated on whether participants had difficulties with AWARE. Open-ended feedback, if provided, was reviewed and key themes and quotes are included as examples (note: below or in supplement).

### Smartphone-Based Mobile Sensing: Application Data Extraction and Processing

To process high-dimensional raw data and extract specific behavioral features (i.e., digital phenotyping), the Reproducible Analysis Pipeline for Data Streams (RAPIDS) was used (Vega et al., 2021). Part of digital phenotyping data cleaning process includes removing times when AWARE might not have worked properly in certain time periods. As part of processing, the first decision to be made is regarding time bins for extracted data. In this study, hourly bins were selected to capture behavioral features of application use (and other sensors) in any given hour across the entire study period per participant. The choice for hourly bin was based on the theoretical constructs of interest and their ability to be explored on an hourly basis given the length of the study (31 days). However, different time bins may yield different results, and warrants further examination in future research. Determining time bins is needed to distinguish between missing data points due to 0.00 min of application use in a given hour (i.e., participant not using social media application in that hour) or AWARE not running properly for most of that hour (e.g., low battery mode on, AWARE crashed, etc.). For this study, we considered any hour with at least 30 min of AWARE data to be a valid hour in which 0.00 min of application use was considered to reflect actual app use; app use was marked as “NA” for hours during which AWARE was running for less than 50% *and* for which no app usage was captured.

#### Phone Data Yield

The phone data yield uses data from multiple sensors to extract data yield features to calculate the ratio between the number of valid minutes and the duration in minutes of a time segment. Within our hourly (60-min) time bins, the data yield ratio provides information about how much of each hour that AWARE was successfully running and collecting data from the participants’ phone. For example, a valid minute is any 60-s window when any phone sensor logged at least 1 row of data across active sensors. This is used as a metric of feasibility of using AWARE, with a high data yield, on average and per participant, indicating the percentage of time that AWARE was successfully collecting data. Average scores and percentages of data yield across the study and individual participants were calculated. Of note, data yield ratio cannot be obtained for specific sensors. This is in part because, for a sensor like applications foreground, for which data are only collected on an event-related basis (e.g., any time the participant launches an app), a measure of data yield would be relatively uninformative. For example, a value of 0 for applications foreground-specific data yield for a particular time segment could either reflect that AWARE was open and functioning but the participant was not engaging with any apps, or that the participant was engaging with apps but AWARE was closed or not functioning and thus those data were not captured.

#### Objective Social Media Categorization

The ‘application foreground’ sensor was used in this study to capture individual features for each application used by participants in this study. Of specific interest was extracting social media data, which included examination of specific application use features or summarized within different categories. Categories were summarized automatically by the Google Play store. However, we also examined differences in categorization based on the Pew Research Center’s recent definition of “popular social media” (Anderson et al., 2023) and commonly used academic definitions of social media. Analysis of multiple methods of categorization was done to highlight how human decision making may influence analysis and findings associated with these data (Carr & Hayes, 2015, p. 51). Individual applications captured with the application foreground sensor include the genre categorization defined by the Google Play Store. These apps are determined a priori by Play Store and included in a Play Store-defined social media category (see Table 1).

**Table 1:** Definitions of social media categories

Social Networking Sites (SNS)	Broad Social Media	Google Play	Popular Social Media
Primary goal is asocial networking and includes Social Networking Sites (SNS) allowing for public & private, asynchronous & synchronous, and a range of user-generated content), and Community/Affinity sites (mainly public, asynchronous, and user-generated content). In this definition, apps have potential broader appeal (even if used for one purpose by one individual vs. the app itself is only used for one specific purpose).	Primary goal is social networking, but more broadly defined as including apps that only have profiles (and no asynchronous interaction, such as messaging, video social networking apps—still including public potential) and apps that allow social networking for one specific purpose (e.g., Video Sharing).	Applications defined by the Google Play store as social media	Applications defined by Pew Research Center as the most popular social media applications in 2024

*Note*. Both SNS and Broad social media categories also needed to meet the following criteria: “Internet-based channels that allow users to opportunistically interact and selectively self-present, either in real-time *or* asynchronously with both broad and narrow audiences who derive value from user-generated content and the perception of interaction with others” - Carr & Hayes, 2015 (p. 51).

Thus, each SM feature must include:

1) User-generated content (typically that allow for self-presentation via text, photo, videos, etc).

2) Asynchronous opportunity for interaction (which allow for opportunistic interaction, or the perception of interacting) (i.e. liking, commenting) (interact with content that is posted, not just messaging ‘asynchronously’ or looking through profiles ‘asynchronously’)

3) Opportunity for social networking with public audiences

Social media was broadly defined as “Internet-based channels that allow users to opportunistically interact and selectively self-present, either in real-time or asynchronously with both broad and narrow audiences who derive value from user-generated content and the perception of interaction with others” (Carr & Hayes, 2015, p. 51). Based on this definition, social media applications were coded by the research team based on specified criteria related to application features. Social media features included apps that had to provide *opportunities* for: (1) users to generate content (share text, videos, photos, profiles) within networks, (2) public networking or interaction, and (3) asynchronous interaction (content or users can be interacted with at any time) or synchronous interaction (or perception of interaction). Private networking could also exist within apps; however, applications that did not include opportunities for public networking were not included (e.g., private group messaging tools, video calls, etc.). Given the multifunctionality of applications and the potential for social networking within many apps, we created two definitions of social media for analysis.

##### Social Networking Category

The first category of ‘social networking’ apps was defined as online platforms where people engage in social relationships or social networks, where they can both create, share, and view user-generated content synchronously (in real time) and asynchronously (scroll/view without other present), as well as interact with others in personal and public spaces (e.g., direct message others AND post/interact in groups or posts). In these platforms, users can typically like, share, follow, comment on posts and follow other users, and multimedia content can be created or shared (e.g., photos, text, videos). Common social networking apps include Facebook, Instagram, Snapchat, or X/Twitter. In addition, the narrower social media category also included ‘online community/affinity groups or forums’, defined as platforms where people connect around interests/groups, where they can both create, share, and view user-generated content, as well as interact with others in public spaces (post/interact in groups or posts) or direct message. Common examples include Tumblr, Reddit, and Discord. Given considerable overlap between the aforementioned categories, and evolving functions of applications, any application that fit within these categories were defined and coded as ‘social networking.’

##### Broader Social Media Category

The second, broader social media category included apps that met criteria for ‘social networking’ (defined above) and additional platforms or apps that allow users to create and share user-generated content (photos, asynchronous videos, or live streaming) without direct/private messaging (e.g., Vimeo or Twitch), with synchronous interaction with public users as the primary user function (e.g., Omegle), or some functions of social networking for specific purposes versus broader network potential (e.g., StoryFire). Note that the apps for video chatting with only private capacities (e.g., FaceTime) were not included in either social media category. Apps that were primarily for social networking of gamers (e.g., Twitch, Plink) also were included in the broader social media category, while applications that were primarily gaming apps with a social component (e.g., Fortnite) were not included in this study as social media, but have the potential for further coding and categorization in future analysis.

##### Popular Social Media

A “popular social media” category was defined as applications included in the annual Pew Research Center Survey (Anderson et al., 2023), which includes: TikTok, Instagram, Snapchat, Facebook, YouTube, Reddit, Discord, and Twitch. Although WhatsApp has traditionally been included in survey polls, it is a private messaging app that does not meet the above definition of social media and therefore, was not included in this category. BeReal was not available at the time of data collection and is therefore not included in the current categorization, though should be included in future popular social media categorization categories.

##### Social Media Coding Procedures

Based on these definitions, five independent coders (including undergraduate, graduate, and faculty) reviewed the list of unique applications used by study participants and independently coded the applications. Consensus meetings were held to discuss apps where categorization (broad vs. narrow) was not completely agreed upon in order to resolve their categorizations. These were generally apps with ambiguity between synchronous and asynchronous interaction. To clarify the difference between our broad and narrow definition of social media, apps with the opportunity for social networking with public audiences (for example, follow unknown users, sharing content with unknown users), asynchronous interaction (beyond messaging or looking at profiles; the ability to scroll, watch, comment, like, etc. content at any time), and user-generated content. The features of the apps without full agreement were reconsidered and closely compared to the broad vs. narrow definitions until reviewers reached a consensus. Disagreement among coders was particularly notable for the narrow category of social networking, where some apps (e.g., YouTube) can be considered to be a primary video sharing app or social networking tool, but is uniformly agreed upon to be ‘social media’. Note that these definitions and categories were developed in discussion and collaboration with our ongoing youth advisory board of high school students (N = 16). See Table 1 for each category definition.

#### Behavioral Feature Extraction of Social Media

Mobile sensing data collected using AWARE was processed using the Reproducible Analysis Pipeline for Data Streams (RAPIDS; Vega et al., 2021), which is a digital phenotyping tool used to process the raw data and extract higher-level information. Behavioral features that can be extracted from RAPIDS for application sensor data can be found in Supplementary Table 1 and the RAPIDS website (https://www.rapids.science/1.9/features/phone-applications-foreground/). Our team examined the “sum duration” and “count event” features; these two features most closely align with prior literature on social media (i.e., “social media screen time/duration” and frequency of checking). Importantly, “count event” is more appropriate than count episode. Specifically, count event features count the unique number of times an adolescent opened an application in each time bin (e.g., each hour). On the other hand, count episode features detects only whether or not an adolescent opened a given application during a given time bin. Future research may seek to use other metrics (e.g., max duration) to focus on periods when adolescents use social media most. Other features, such as “frequency entropy,” which measures the breadth of applications used within a given period, may be of interest moving forward, but interpretability at the current time limits its immediate applicability. This approach aligns with the concept of digital phenotyping, which involves using digital data to measure and analyze behavioral patterns and mental health. As described above, these data may also be paired with subjective intensive monitoring data (e.g., experience sampling data) to obtain an in-depth picture of adolescents’ social media use behaviors and mental health symptoms on a momentary timescale. In this study, we focused on hourly data (as described above) and daily social media use features (within a 24-h period captured by the same date: 00-23:59). However, breakdown of intensively captured mobile sensing data remains an important point of consideration the research question and individual differences in sleep-wake rhythms.

## Results

### Participant and Study Characteristics

Table 2 contains demographic information for the current study sample. The sample consisted of 19 participants. The sample was majority white (78.95%) and boys (68.42%). The average number of days participants remained in the study was 31 days. All participants except one were in the study for at least 26 days, with one participant withdrawing from study procedures after 15 days.

**Table 2:** Sample demographics

	N (%)
Age, M (SD)	15.84 (1.01)
Race	
White	15 (78.95%)
Black/African American	2 (10.53%)
More than one race	2 (10.53%)
Ethnicity	
Hispanic/Latine	0 (0%)
Non-Hispanic/Latine	19 (100%)
Sex	
Male	13 (68.42%)
Female	6 (31.58%)
Gender	
Boys	11 (57.89%)
Girls	7 (36.84%)
Non-binary/third gender	1 (5.26%)
Transgender	2 (10.53%)
Sexual Orientation	
Heterosexual/Straight	14 (73.68%)
Bisexual	3 (15.79%)
Queer	1 (5.26%)
Bi-curious	1 (5.26%)
SES - Society, M (SD; Range)	6.37 (1.5; 4-9)
SES - School, M (SD; Range)	6.53 (1.74; 4-10)

*Note*. *M*, Mean; *SD*, standard deviation; *Latine*, a gender-neutral term for Latino/Latina; *SES – School*, socioeconomic status relative to society at-large; *SES – School*, socioeconomic status relative to others in the same school; for both SES measures, 1 = much lower SES relative to these groups (society or school), and 10 = much higher SES relative to these groups; total N = 19.

### Acceptability: How Acceptable was the AWARE Application with Teens?

Adolescent participants reported a median of 1 “not at all” to 2 “sometimes” in terms of how often they had concerns about their privacy (M = 1.16, SD = .50; range = 1–3), had issues with AWARE like application crashes (M = 1.68, SD = .89, range: 1–4), and perceived that it drained their battery (M = 1.26, SD = .56, range: 1–3). In addition, most participants reported they “often” forgot that it was installed on their phones (M = 3.21, SD = .02, range: 1–4). A total of 11 participants provided additional open-ended feedback about any AWARE app issues. There also were minimal additional feedback noted by teens, though a total of seven participants noted the app spontaneously closed or crashed occasionally (but most stating it was easy to reopen and resume app), one participant stating they often closed out of the app in the beginning (which pauses data collection), and one participant noting that AWARE drained their battery faster because their Android phone did not have a good battery. No privacy concerns were noted in the open-ended feedback.

### Feasibility: How Much Data did we Get from Teens?

In total, there were 10,038 hourly observations collected from the ‘application foreground’ sensor across all participants from social media apps. The study had a 74.18% data yield ratio, ranging for each individual from 34.8 to 99.9% (only one participant had < 50%). This data yield ratio indicates that AWARE was functioning throughout the study for most adolescents. This is generally a higher proportion of data than one would expect to get from relying on self-report and also more data generated via hourly than a daily self-report. However, with a 74% data yield, there is still room for improvement with potential to get a higher data yield ratio in studies utilizing the application sensor of mobile sensing applications.

### Objective Social Media Use: How do we Define and Describe Social Media Use?

#### Defining Social Media

Overall, there were 645 unique applications used across the study participants. There was general consensus among coders in determining the social media application categories, with the broader social media category having high agreement (Kappa = .97) and a relatively high agreement for the narrower definition of ‘social networking’ (Kappa = .92). A total of total of 41 apps met criteria for the broader ‘Social Media’ category and 20 applications met criteria for ‘Social Networking’. A total of 26 apps were a priori defined as social media by the Play Store, which notably did not include many popular social media apps (e.g., YouTube was not included), and some social media apps instead categorized as ‘lifestyle’, ‘videoplayerseditors’, or ‘unknown’. A total of 9 applications were included in the Popular SM Category, defined by the Pew Research Institute (Gottfried, 2024). That said, given our study collected data in 2020 (four years prior to Pew’s recent report), not all aforementioned apps were used by our study sample. Specifically, Linkedin was not used by any participants and BeReal had not yet been released. We chose not to include WhatsApp because the function of the app (messaging and calling) was not consistent with our definition of social networking or social media. See Table 3 for a breakdown of apps within each category.

**Table 3:** Complete key of social media applications and categories

	App	Package Name	SNS	Broad	Google Play	Popular SM
1	ACM	com.narvii.amino.manager	x	x	x	
2	Amino	com.narvii.amino.master	x	x	x	
3	BIGO LIVE	sg.bigo.live	x	x	x	
4	byte	co.byte	x	x	x	
5	Facebook	com.facebook.katana	x	x	x	x
6	Hoop	com.dazz.hoop	x	x	x	
7	Instagram	com.instagram.android	x	x	x	x
8	Litmatch	com.litatom.app	x	x	x	
9	LMK	com.lightspace.lmk	x	x	x	
10	Peanut	com.teampeanut.peanut	x	x	x	
11	Quack	com.quack.app	x	x	x	
12	Reddit	com.reddit.frontpage	x	x	x	x
13	Snapchat	com.snapchat.android	x	x	x	x
14	TikTok	com.zhiliaoapp.musically	x	x	x	x
15	Tumblr	com.tumblr	x	x	x	
16	Twitter	com.twitter.android	x	x	x	x
17	Discord	com.discord	x	x		
18	Quora	com.quora.android	x	x		
19	rif is fun	com.andrewshu.android.reddit	x	x		
20	YouTube	com.google.android.youtube	x	x		x
21	Joi	video.chat.joi		x	x	
22	Monkey	cool.monkey.android		x	x	
23	Pinterest	com.pinterest		x	x	
24	Plink	tech.plink.PlinkApp		x	x	
25	Weverse	co.benx.weverse		x	x	
26	Wink	co.ninecount.wink		x	x	
27	YOLO	com.popshow.yolo		x	x	
28	Yubo	co.yellw.yellowapp		x	x	
29	iFunny	mobi.ifunny		x		
30	Likee	video.like		x		
31	LMKpoll	com.rubysparklabs.lmk		x		
32	Omegle	com.ni.Omegle		x		
33	sendit	com.fullsenders.sendit		x		
34	Spotafriend	com.mylol.spotafriend		x		
35	Steam	com.valvesoftware.android.steam.community		x		
36	StoryFire	com.storyfire.storyfire		x		
37	Tinder	com.tinder		x		
38	Twitch	tv.twitch.android.app		x		
39	Vimeo	com.vimeo.android.videoapp		x		
40	Vine Camera	co.vine.android		x		
41	Wattpad	wp.wattpad		x		
42	Airtime	com.signal.android			x	
43	BAND	com.nhn.android.band			x	
44	Houseparty	com.herzick.houseparty			x	

*Note*. *SNS*, Narrow Social Networking Category; *Broad*, Broad Social Media Category; *Google Play*, Google Play Store-Defined Social Media Category; *Popular SM*, Popular Social Media Category defined by Pew Research Center

#### Extracted Social Media Behavioral Features

Given the complexity of defining social media, descriptives of duration and checking frequency for each defined category were first examined. Table 4 includes the average daily sum and checking frequency across all hourly observations (N = 10,038), study days (N = 564), and people means (N = 19) for each social media category. Average daily sum duration (95 min) and checking frequency (160 counts) was highest for the broader social media category, which included the 41 possible apps. Social networking and broader social media categories (both coded by research team) were fairly similar to one another, with minor differences of 2–3 min per day and 6 checks per day, despite a difference of 21 additional applications coded within the broader social media category. However, social networking and popular social media categories also were largely similar (despite difference of 11 applications in social networking), with average differences of 5–6 min per day and 9 checks per day. Of note, Play Store-defined social media category was the lowest in daily sum duration (60 min) and checking frequency (123 checks), and least similar to the other three domains by about 30 min per day (sum duration) and at least 20 checks per day.

**Table 4:** Averages of social media use (duration and checking) by social media category

Social Media Feature	Broader Social Media	Social Networking	Popular Social Media	Play Store-defined
Between-Person Daily Sum, M(SD), range (N = 19)	94.84 (62.47), 6.71–205.83	92.66 (60.81), 6.62–205.76	87.16 (59.62), 6.62–66.99	59.68 (53.98), .43–198.65
Between-Person Daily Checks, M (SD), range (N = 19)	159.97 (125.3), 8.17–537.48	153.67 (115.51), 7.97–476.07	144.81(115.78), 7.96–466.62	123.18 (120.68), 1.1–459.52
Average Daily Sum, M (SD), range (N = 564)	98.05 (79.34), 0–405.87	95.94 (77.65), 0–379.47	89.92 (75.27), 0–352.30	61.90 (65.3), 0–369.29
Average Daily Checks, M (SD), range (N = 564)	164.15 (146.30), 0–1103	158.37 (134.00), 0–931	148.58 (131.76), 0–931	125.71 (138.76), 0–1018
Average Hourly Sum, M (SD), range (N = 10,038)*	5.51 (9.11), 0–60	5.39 (9.01), 0–60	5.05 (8.76), 0–60	3.48 (6.94), 0–60
Average Hourly Checks, M(SD), range (N = 10,038)*	9.22 (14.21), 0–135	8.90 (13.44), 0–116	8.35 (12.93), 0–115	7.06 (12.57), 0–135

Note: * does not take into account hours when individuals were asleep or not using their phone

**Fig. 1:**
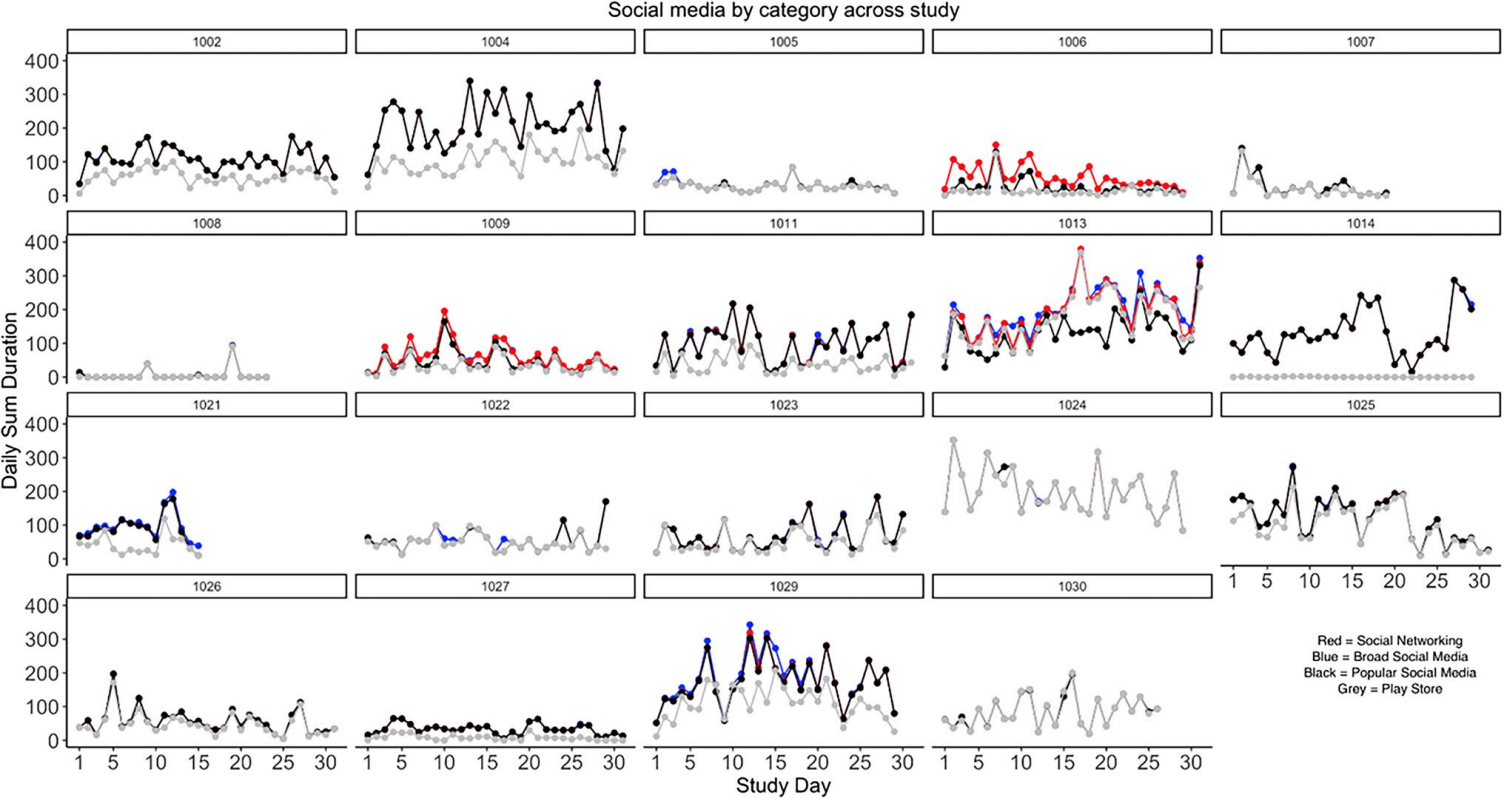
Daily time spent on social media by category, across participants

**Fig. 2:**
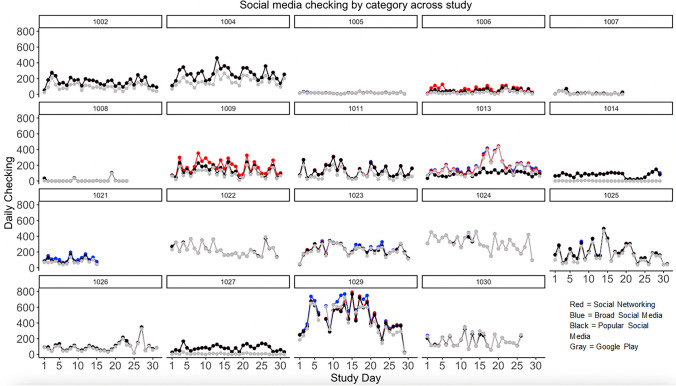
Daily checking frequency by category, across participants

Within-person correlations of daily social media use duration by social media categories (calculated using the rmcorr package in R) were highest for coded categories of broader social media and social networking (*r =* .99), though remained high with popular social media for broad social media (*r =* .95) and social networking (*r =* .96). Within-person correlations were lowest for all categories with Playstore-defined social media (*r* = .72–.79). Results were similar for checking frequency, though correlations with Playstore-defined social media were higher for this category (*r=* .90–.93). Results suggest that individuals who used and checked social media more frequently were relatively consistent across categorizations. Figures 1 and 2 visually present the daily sum and checking frequencies for each category and each participant in the study. Of note, some individuals may appear to only have two categories given minor differences (at this scale) in duration/frequency across broader social media, social networking, and popular social media categories. However, some individuals have more distinctive characteristics based on how social media is defined, as reflected in substantial differences in social media duration and frequency within the same day (See Figs. 1 and 2). Thus, for some individuals, the popular social media category may best capture individuals’ daily usage and frequency of checking behaviors, whereas there may be more pronounced individual differences in social media for less popular or commonly used social media platforms (that may only be captured by more thorough analysis).

Social media use category averages (between-person, daily, or hourly) fail to account for the significant individual variability observed *within* person. Intraclass correlations suggested that participants had more between-person variability for daily sum duration, ranging from 60% (social networking, broad social media, and popular social media) to 68% for Play Store-defined social media. Similarly, most variability for social media checking occurred between-person (66.68–70.28%), with broad social media having the most within-person variability. To visually present the considerable variability within-person and day-to-day, Figs. 1 and 2 depict the daily social media use duration and checking by study day across all participants in the study (for different categories). Of note, the amount of time adolescents spend on social media and number of times social media was checked in a day were only moderately correlated (*r =* .52–.59) across categories.

#### Not All Popular Social Media Apps are Created Equal

All teens (100%) used YouTube at some point in the study, with Snapchat and Instagram as the second and third most popular social media apps, respectively, followed by TikTok and Discord. On average, teens actively used 8 (SD = 3) social media apps at any time in the study, including apps within any social media categorization (i.e., social networking, broader social media use, popular social media, or Play Store-defined category). A total of 41 social media apps were used by participants in the study, though this reflected 19 different individuals.

**Fig. 3:**
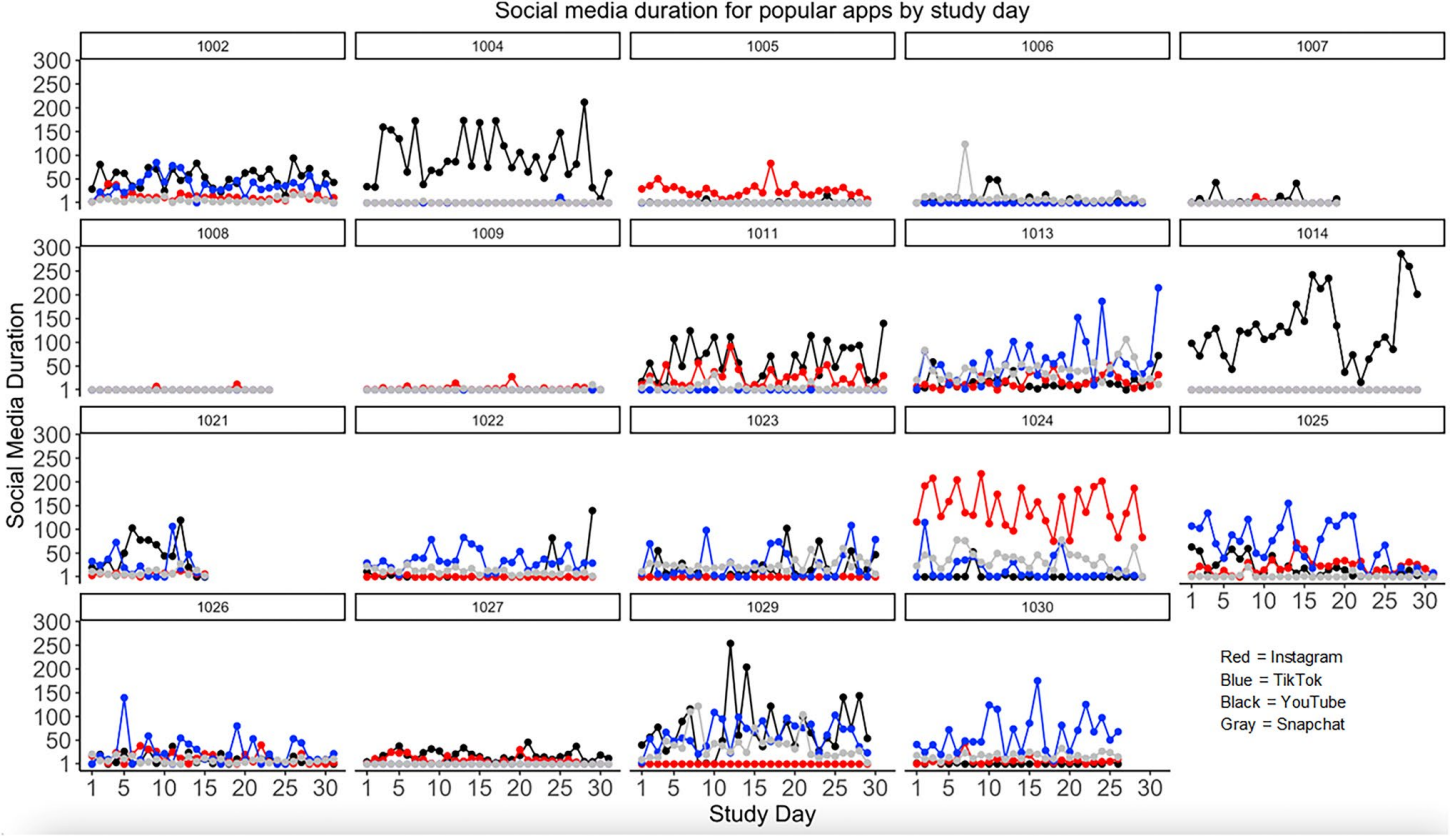
Daily time spent on popular social media apps

**Fig. 4:**
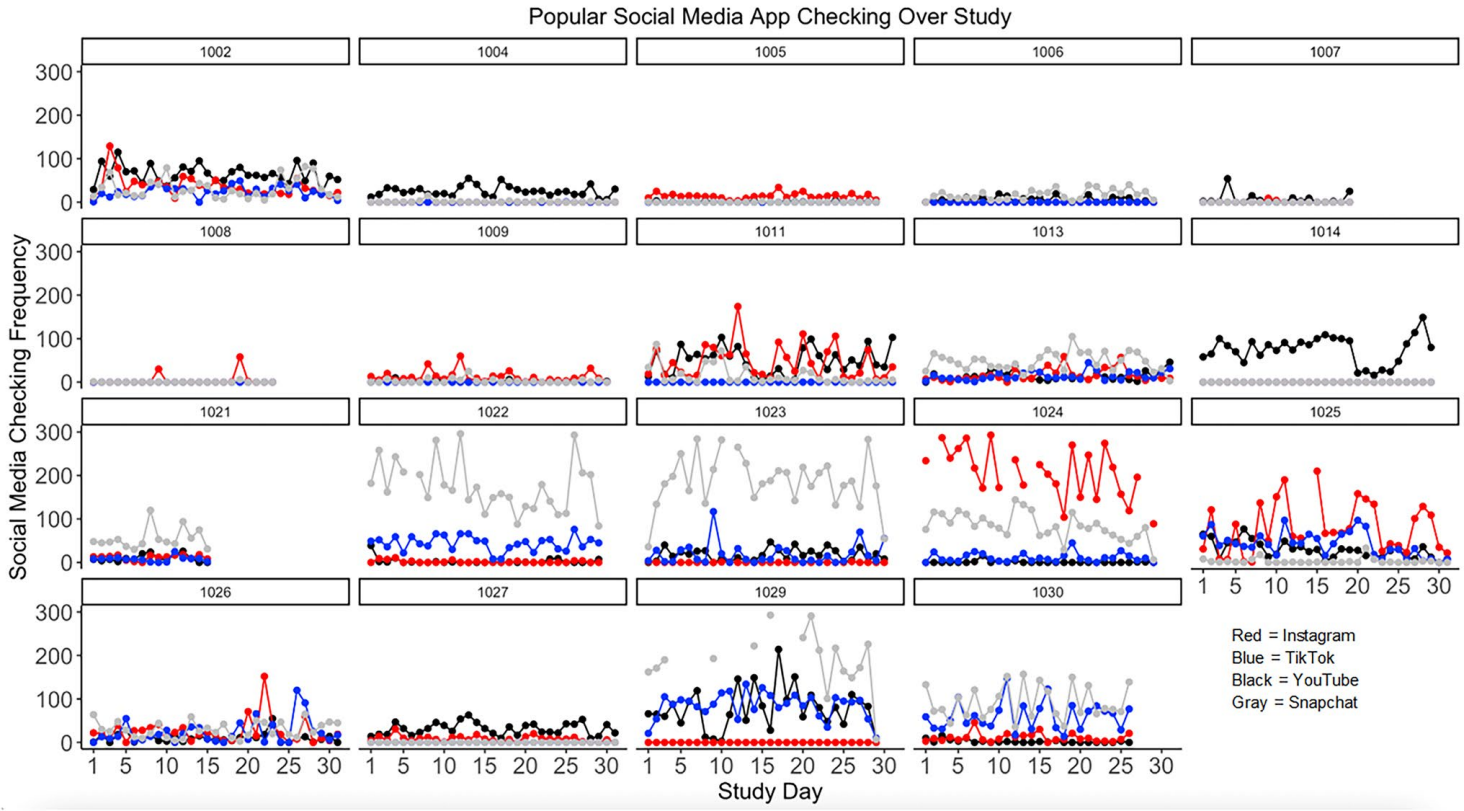
Daily checking frequency of popular social media apps

Despite similarity of social media categories, there are individual differences in how much social media apps were used within category. In terms of popular social media applications, YouTube was the only social media app used by all participants. Averaging across all participants, adolescents used YouTube the most on average per day based on daily sum duration (M = 30.11 min; SD = 46.24, range = 0–286.82 min). Yet, Snapchat was the most frequently checked social media app (M = 53.64 checks; SD = 91.82, range = 0–730) across all participants. Taking into account that only YouTube was used by all participants, means also were calculated based on number of participants who used the app. With this in mind, TikTok was the longest used by participants (N = 11; sum duration mean = 36.72; SD = 21.24; 0.36–62.45), whereas Snapchat remained the most checked (N = 16; checking mean = 65.31; SD = 87.69; .10–304.45). Table 5 includes descriptive information of usage for individual social media apps. In addition, Figs. 3 and 4 present the four most commonly used social media apps in this study (and based on Pew Research Center survey data; Anderson et al., 2023), which highlights individual differences (and daily fluctuations) in *which* apps were being used the most over the study period.

**Table 5:** Descriptive usage information of each social media application

	App	N	%	Average Daily Minutes	SD Daily Minutes	Average Daily Checks	SD Daily Checks	Category
1	YouTube	18	94.74	30.75	36.95	23.28	25.65	SNS, Broad, Popular SM
2	Snapchat	16	84.21	12.77	13.86	65.31	87.69	SNS, Broad, GP Popular SM
3	Instagram	15	78.95	18.83	36.01	32.89	57.29	SNS, Broad, GP, Popular SM
4	Discord	11	57.89	3.28	4.19	14.69	20.95	SNS, Broad
5	TikTok	11	57.89	36.72	21.24	29.52	25.49	SNS, Broad, GP, Popular SM
6	Twitter	7	36.84	18.31	30.14	38.40	56.12	SNS, Broad, GP, Popular SM
7	Facebook	6	31.58	1.72	2.35	1.93	1.51	SNS, Broad, GP, Popular SM
8	Reddit	6	31.58	6.20	6.78	4.58	5.04	SNS, Broad, GP, Popular SM
9	Twitch	6	31.58	0.12	0.20	0.24	0.33	Broad
10	YOLO	5	26.32	0.10	0.08	1.74	1.82	Broad, GP
11	Pinterest	4	21.05	3.63	6.13	3.34	3.89	Broad, GP
12	Hoop	3	15.79	0.57	0.72	3.64	4.13	SNS, Broad, GP,
13	Steam	3	15.79	0.10	0.09	0.12	0.07	Broad
14	Amino	2	10.53	25.01	35.07	37.49	52.30	SNS, Broad, GP
15	Houseparty	2	10.53	0.15	0.13	0.58	0.45	GP
16	iFunny	2	10.53	5.79	4.85	12.06	16.03	Broad
17	LMKpoll	2	10.53	0.12	0.17	1.10	1.51	Broad
18	Quora	2	10.53	0.47	0.52	0.47	0.31	SNS, Broad
19	rif is fun	2	10.53	24.26	9.05	38.32	22.45	SNS, Broad
20	Wink	2	10.53	3.72	3.99	23.88	26.60	Broad, GP
21	Yubo	2	10.53	1.69	2.32	9.32	12.86	Broad, GP
22	ACM	1	5.26	1.23	NA	1.69	NA	SNS, Broad, GP
23	Airtime	1	5.26	0.16	NA	0.62	NA	GP
24	BAND	1	5.26	0.01	NA	0.07	NA	GP
25	BIGO LIVE	1	5.26	0.01	NA	0.21	NA	SNS, Broad, GP
26	byte	1	5.26	0.01	NA	0.07	NA	SNS,Broad, GP
27	Joi	1	5.26	0.02	NA	0.17	NA	Broad, GP
28	Likee	1	5.26	1.63	NA	0.45	NA	Broad
29	Litmatch	1	5.26	0.07	NA	0.24	NA	SNS, Broad, GP
30	LMK	1	5.26	0.11	NA	0.24	NA	SNS, Broad, GP
31	Monkey	1	5.26	0.09	NA	0.13	NA	Broad, GP
32	Peanut	1	5.26	0.003	NA	0.07	NA	SNS, Broad, GP
33	Plink	1	5.26	0.64	NA	1.47	NA	Broad, GP
34	Quack	1	5.26	0.15	NA	0.38	NA	SNS, Broad, GP
35	sendit	1	5.26	0.01	NA	0.03	NA	Broad
36	Spotafriend	1	5.26	0.001	NA	0.02	NA	Broad
37	StoryFire	1	5.26	0.005	NA	0.03	NA	Broad
38	Tinder	1	5.26	0.11	NA	0.34	NA	Broad
39	Tumblr	1	5.26	1.75	NA	1.98	NA	SNS, Broad, GP
40	Vimeo	1	5.26	0.0003	NA	0.02	NA	Broad
41	Wattpad	1	5.26	0.22	NA		NA	Broad
42	Weverse	1	5.26	0.01	NA	0.11	NA	Broad, GP

*Note*. *SNS*, Social Networking; *Broad*, Broad Social Media Category; *GP*, Google Play-Defined Social Media; *Popular SM*, Popular Social Media, defined by Pew Research Center

## Discussion

Smartphone-based mobile sensing provides unique opportunities to capture objective social media use to better understand how and when teens are using social media and its potential relationship to mental health. In this study, we found that teens generally reported that mobile sensing procedures and the smartphone application were acceptable, with minimal concerns noted about privacy, battery usage, or application crashing/errors logged. In general, AWARE collected data from teens’ phones 74% of the time that they were enrolled in the study, which indicates its potential feasibility of use for teens. Importantly, smartphone-based mobile sensing also provided key insights into adolescents’ social media use, including the breadth of potential social media applications and patterns of use, which advances current approaches to studying social media use. While mobile sensing provides unique opportunities to capture objective application use on teens’ smartphones, there are key conceptual and technical challenges to be addressed, including the definition and categorization of social media applications, decision points for both processing and analyzing this high-dimensional data, and technical considerations limiting its scalability.

### Challenges: Defining Social Media, Processing Data, and Technical Considerations

#### How do we Define Social Media?

What constitutes social media is both ambiguous and rapidly evolving as social media shifts and transforms and new technological features emerge. While the boundaries of traditional social media platforms were previously more defined (e.g., Facebook), many applications now feature social components inherent in the design and social gaming platforming include interactive points of connection and opportunities for self-presentation and expression. In many ways, how social media is defined depends on the research question and study goals. In this study, a total of 41 applications were defined as social media. However, despite the large number of possible social media apps, the popular social media category was relatively similar to both the social networking and broader social media coded categories for most teens—suggesting that the nine popular social media apps may be driving estimates in these overall categories. Given the time-intensive approach of coding all social media applications, focusing primarily on the popular social media apps (defined by Pew Research Center and Common Sense; Anderson et al., 2023) as a category and individual applications therein may be the most efficient approach to apply mobile sensing for examining adolescents’ social media use.

However, the broader inclusion criteria of social media (as coded by social networking and broad social media categories) also may yield meaningful distinctions for some teens, capturing less popular or commonly used apps that are still important to consider within individualized patterns of social media use that otherwise would have gone unrecognized. This is particularly important when considering how teens with a range of sociodemographic identities may use social media (Charmaraman et al., 2022), which is important to be further explored.

In contrast, Play Store-defined social media was the least similar to our conceptually defined and popularly defined social media categories. In our study, the Play Store defined 26 apps as ‘social’, but missed others that are considered social media (e.g., YouTube). Importantly, this led to considerable differences in the total estimated duration of social media use per day and checking frequency, particularly given that YouTube was the most used social media application by adolescents in our study, consistent with Pew Research Center data (Anderson et al., 2023). Thus, relying solely on the a priori categorizations by Google (and potentially iOS categorizations) may inaccurately reflect the range and use of social media by most adolescents.

While we determined ‘social networking’ and ‘broader social media’ categories, there are other ways in which social media could be grouped. For instance, social media apps that are highly visual or video-based may be relevant to examine, or based on individual features within social media (e.g., permanence, asynchronicity, publicness, etc.; Nesi et al., 2022). Furthermore, applications used for social networking primarily by gamers (e.g., Discord, Steam, twitch) were included in the Broad Social media category, but we did not separately examine social gaming apps (which are to be coded separately). Gaming apps may yield different patterns use and potential links with mental health (Kowal et al., 2021; Snodgrass et al., 2011); social gaming apps might have unique connections to mental health, especially considering benefits of social connection online (Hamilton et al., 2020a, 2020b), but research on specific effects of social gaming is limited. Another major challenge in defining social media lies in the rapid changes within social media platforms, with new functions and features. Thus, it is possible that some of the applications included in these categories may shift in their functionality over time, making it important to re-categorize these applications on an ongoing basis and with input from teens with diverse identities.

#### How do we Process High-Dimensional Data?

In addition to defining social media, there are multiple decision points that must be made about how to process and extract the behavioral data. First, the decision to bin mobile sensing data hourly may come with a loss of a more fine-grained perspective of what may occur *within* an hour. This choice may impose human judgment on the data, though given the length of the study duration (one month), hourly bins were determined to provide sufficient nuance within the data with the possibility of 720 hourly bins per participant (24 h × 30 days). Second, for ease of presenting the data, data also was summed within a 24-h period (00-23:59), which does not capture adolescents’ social media patterns within specific time bins or across the day. Examining social media use patterns may lead to specific insights for sleep (Hamilton et al., 2020a, 2020b; Hamilton, Jorgensen, et al., 2023a, 2023b; Scott et al., 2019) and potential points of intervention for those who may be excessively using or checking social media during nighttime hours. This is a critical point for future research using these methods, as sensing data provides one of the only opportunities to capture times adolescents may be using social media use patterns without researcher intervention that could interfere with use and sleep (e.g., sending EMA prompts at nighttime).

#### How do Technical Considerations Affect Scalability of this Approach?

Another important consideration of this work is that this method is currently only available with Android phones, which limits comparison to iOS objectively captured data. However, newer partnerships with Apple may allow for similar approaches to be taken with both Android and iOS users (Langholm et al., 2023), broadening the potential scope and application of this work. Thus, it is important to note that participants’ whose applications were unable to monitor their usage or were iPhone users had no social media data recorded during the study period and were removed from data analysis. There are additional issues that may affect access to application sensor data, including missingness due to parental security applications, low battery mode, or other factors. For instance, some parental security applications interfered with data collection, in that this regularly needed to be monitored and discussed with parents and teens as part of the consent and assent process. Depending on the length of the study, this limitation may reduce willingness of parents to have their adolescents participate. In addition, decisions need to be made regarding potential missing social media data (i.e., NA vs. 0;). One larger issue is that it is not always clear *why* there is missing data in the sample, which could be due to no social media use during that time, or the phone stopped collecting data. In other words, researchers need to determine whether a missing data value reflects *true* missing data (i.e., AWARE was not running properly) or whether a participant did not use or check their phones during a given time bin. RAPIDS output provides a data yield for each time bin, demonstrating how long AWARE was running. However, there are many times where, for example, AWARE may be running for part of a time bin (e.g., 40 min out of an hour). If the dataset shows a participant did not use social media during that hour, it may be because they did not use it *or* it may be that they used it during the 20-min time that AWARE was not properly running. In our research studies, we have opted to set a threshold of 50% data yield to label a missing data value as “0.” In other words, if AWARE was running for 30 min or more out of an hour and the data reflect no social media use, we code that as participants not using social media. If AWARE was running for less than 30 min in an hourly period and there is no data for that period, we code that as a true missing value (NA), assuming AWARE may not have captured that data. No matter the threshold researchers set, this necessarily introduces some bias.

### Opportunities: What can we Learn about Teens’ Social Media Use to Inform Clinical Science?

#### Accurately Capturing Social Media Use

There are several unique advantages of using mobile sensing to objectively capture social media use, which is central in furthering the rigor of the science investigating social media and mental health. First, in contrast to self-reported approaches of “typical social media use”, mobile sensing provides unique information about *which* social media applications teens were actively using in our study. Using this approach, our study identified a total of 41 possible applications that could be considered social media based on current social media definitions and features (cite above). Most research has relied on vague definitions of “social media” in self-reported surveys, which likely varies by adolescents’ use and perception, as observed in the current study. Similarly, using only researcher-defined social media apps within such questionnaires or content examination within only 1–2 platforms may obscure individual uses and patterns of social media. For instance, certain social media apps were rapidly expanding at the time of this study (e.g., TikTok), which might have been missed through self-reported methods capturing only well-known types of social media platforms (e.g., Instagram, Snapchat). Objective sensing data also allowed us to better capture the full range of potential social media applications (across categories) without solely relying on a priori definitions or researcher-defined social media apps for self-reported estimates, which would have likely missed the emergence of important and highly used applications. There also were culturally relevant social media applications used by teens in our small sample, including several platforms more popular in other countries (e.g., Wechat). Mobile sensing offers the unique opportunity to capture all applications as they emerge and are used at an individual level, which may lead to important distinctions in how different teens use social media (e.g., belonging, community) and its impacts on mental health. Of note, in this study, we focused on smartphone-based mobile sensing, which may have limited collection of other social media applications used on other devices (e.g., video game consoles, tablets, computers). Thus, other mobile sensing and digital phenotyping procedures to additional higher-level behaviors of social activity across devices would provide a more comprehensive understanding of adolescents’ social media use and behaviors.

#### Potential for Explicating Person-Specific Patterns

In addition, many teens were using multiple platforms of social media over the course of the study, which leads to questions about how and why teens may engage in different social media platforms at different times. There was considerable variability observed of social media by individual and day in the study, highlighting the importance of using more intensive metrics to capture social media use patterns of both duration and checking frequency. In particular, some teens checked social media up to 400 times on some days, even if their overall use was 1–2 h, suggesting that the frequency of checking may impact teens’ perceptions that they use social media ‘almost constantly’ (Anderson et al., 2023). Finally, mobile sensing provides the opportunity to capture both social media use duration and checking frequency at different time intervals (e.g., hourly, daily), and these features may have unique relationships with adolescents’ mental health (Dreier et al., 2024). That said, there are important nuances that could be further explored by future research, including the fact that spending time on or checking different social media apps may vary as a function of the application itself. For example, TikTok and YouTube are video platforms, which adolescents may spend more time on, simply because videos take longer to watch than viewing a picture. An app like Snapchat, for example, may be one that adolescents check frequently, to keep up with messages, but may be one they spend less time on overall, given the nature of the app.

Mobile sensing and digital phenotyping methods yield highly dimensional and rich data that, paired with advanced analytic techniques to harness this data, can provide new insights into a range of adolescents’ behaviors at an idiographic level. Until recently in psychology, most research has focused on nomothetic, or group/average-level, trends in behaviors and outcomes. Focusing solely on group-level trends, however, washes away important differences in constructs that vary greatly within-person, like social media use. As observed in this study, social media use varies and fluctuates considerably, not only between individuals, but also within individuals across both hours and days. Accordingly, social media’s relationship to wellbeing also varies greatly within individual (Beyens et al., 2020, 2021; Valkenburg, Meier, et al., 2022a, 2022b). These patterns make logical sense—what constitutes “social media” is broad (discussed above), and most people may be inclined to use it for many purposes. Whereas at one point in the day, someone may decide to catch up with old friends using Snapchat and feel more connected, they may watch videos at another point in the day on TikTok that elicit insecurities. To account for this important nuance, idiographic and digital phenotyping methods can capture these within-person fluctuations in social media use and experiences in relation to higher level behavioral or clinical states (Perlmutter et al., 2024), such as wellbeing (Beyens et al., 2020, 2021; Valkenburg, Meier, et al., 2022a, 2022b), self-esteem (Valkenburg et al., 2021), and mood (Dreier et al., 2024). Idiographic methods have already provided powerful insight in other areas of psychology, such as in suicide and self-injury (Coppersmith et al., 2021), eating disorders (Levinson et al., 2021, 2022, 2023), as well as mood and anxiety disorders (Fisher et al., 2017).

Use of these methods has allowed for the development of evidence-based treatments that are not just tailored to what individuals tend to experience on average, but to the exact symptoms an individual person is experiencing (Fisher et al., 2019; Fisher & Boswell, 2016; Levinson et al., 2021, 2022, 2023). Currently, evidence-based recommendations around social media use rely on group-level patterns and trends (American Psychological Association (APA), 2023). Use of idiographic and digital phenotyping methods can allow for the development of personalized evidence-based recommendations that consider the unique patterns of social media use experienced by each person. In order to conduct these kinds of analyses, however, it is necessary to collect intensive longitudinal data using methods like smartphone-based mobile sensing, to understand how social media use fluctuates dynamically for individual people over both proximal and long-term timescales. In using these statistical techniques, researchers may also choose to pair mobile sensing data with other forms of intensive longitudinal data (e.g., ecological momentary assessment, physiological data collected via smartwatches) and use digital phenotyping approaches to understand higher-level clinical states. Using the plethora of social media use data collected from mobile sensing within and across days, researchers could use these machine learning approaches to better understand *which* aspects of social media use behaviors best predict these clinical outcomes on different time scales (e.g., occurrence of momentary of suicidal ideation or a suicide attempt over a specified time frame). While the current study provided averages across the participants to inform its potential, with a focus on defining social media, idiographic approaches that yield information about individualized patterns of use across hours and days within participants remain an important area of research (e.g., Beyens et al., 2020; Dreier, Low, et al., Preprint; Valkenburg, Beyens, et al., 2022; Valkenburg et al., 2021). Of note, the current study also collected data during the early months of the COVID-19 pandemic, which may represent a distinct picture of adolescent social media use behaviors, although emerging work suggests that behavioral trends from that time period are lingering to the present day (Hamilton, Dreier, et al., 2023a).

## Conclusion

This study provides an initial overview of utilizing smartphone-based mobile sensing to rigorously capture objective social media use among adolescents, and to discuss these challenges, considerations, and opportunities for application to clinical psychological science. There is an increasing shift in the field to understanding social media experiences versus patterns of use (Prinstein et al., 2020). Nonetheless, mobile sensing using smartphones provides rich, objective information that, especially when paired with information about social media experiences, improves the rigor of research on this topic and has potential to advance our understanding of social media use and mental health.

## Supplementary Information

Below is the link to the electronic supplementary material.

Supplementary file1 (DOCX 14 KB)

## Data Availability

Participants did not consent to data availability; however, data analysis code are available upon request.
