# Supplementary material for: Improving the Science of Adolescent Social Media and Mental Health: Challenges and Opportunities of Smartphone-Based Mobile Sensing and Digital Phenotyping
Source: J Technol Behav Sci. 2024 Oct 18;10(2):301–19. doi: 10.1007/s41347-024-00443-5 (PMC12144053; doi:10.1007/s41347-024-00443-5)
Supplement: Supplementary file 1 — Supplementary file1 (DOCX 14 KB) [file 41347_2024_443_MOESM1_ESM.docx]

| Feature | Units | Description |
| --- | --- | --- |
| countevent | apps | Number of times a single app or apps within a category were used (i.e. they were brought to the foreground either by tapping their icon or switching to it from another app) |
| timeoffirstuse | minutes | The time in minutes between 12:00am (midnight) and the first use of a single app or apps within a category during a time_segment |
| timeoflastuse | minutes | The time in minutes between 12:00am (midnight) and the last use of a single app or apps within a category during a time_segment |
| frequencyentropy | nats | The entropy of the used apps within a category during a time_segment (each app is seen as a unique event, the more apps were used, the higher the entropy). This is especially relevant when computed over all apps. Entropy cannot be obtained for a single app |
| countepisode | apps | Number of times a usage episode of a single app or apps within a category were logged. In contrast to countevent, if an app was used across more than one time segment (for example, across more than one 30-minute segment), the countepisode will be one on each time segment instance. |
| minduration | minutes | For a time_segment, the minimum duration an application was used in minutes |
| maxduration | minutes | For a time_segment, the maximum duration an application was used in minutes |
| meanduration | minutes | For a time_segment, the mean duration of all the applications used in minutes |

*Supplemental Table 1*. Features extracted by RAPIDS

*Note*. This table was reproduced from: <https://www.rapids.science/1.10/features/phone-applications-foreground/>
